# BioWardrobe: an integrated platform for analysis of epigenomics and transcriptomics data

**DOI:** 10.1186/s13059-015-0720-3

**Published:** 2015-08-07

**Authors:** Andrey V. Kartashov, Artem Barski

**Affiliations:** Division of Allergy and Immunology, Cincinnati Children’s Hospital Medical Center and Department of Pediatrics, College of Medicine, University of Cincinnati, Cincinnati, OH USA; Division of Human Genetics, Cincinnati Children’s Hospital Medical Center and Department of Pediatrics, College of Medicine, University of Cincinnati, Cincinnati, OH USA

## Abstract

High-throughput sequencing has revolutionized biology by enhancing our ability to perform genome-wide studies. However, due to lack of bioinformatics expertise, modern technologies are still beyond the capabilities of many laboratories. Herein, we present the BioWardrobe platform, which allows users to store, visualize and analyze epigenomics and transcriptomics data using a biologist-friendly web interface, without the need for programming expertise. Predefined pipelines allow users to download data, visualize results on a genome browser, calculate RPKMs (reads per kilobase per million) and identify peaks. Advanced capabilities include differential gene expression and binding analysis, and creation of average tag -density profiles and heatmaps. BioWardrobe can be found at http://biowardrobe.com.

**Electronic supplementary material:**

The online version of this article (doi:10.1186/s13059-015-0720-3) contains supplementary material, which is available to authorized users.

## Introduction

The recent proliferation of next-generation sequencing (NGS)-based methods for analysis of gene expression, chromatin structure and protein–DNA interactions has opened new horizons for molecular biology. These methods include RNA sequencing (RNA-Seq) [1], chromatin immunoprecipitation sequencing (ChIP-Seq) [2], DNase I sequencing (DNase-Seq) [3], micrococcal nuclease sequencing (MNase-Seq) [4], assay for transposase-accessible chromatin sequencing (ATAC-Seq) [5], and others. On the “wet lab” side, these methods are largely well established and can be performed by experienced molecular biologists; however, analysis of the sequenced data requires bioinformatics expertise that many molecular biologists do not possess. Re-utilizing published datasets is also challenging: although authors usually comply with the longstanding requirement to deposit raw data files into databases such as the Sequence Read Archive (SRA) or Gene Expression Omnibus (GEO), it is impossible to analyze these datasets without special expertise. Even when processed data files (e.g., gene expression values) are available, direct comparison between datasets is ill-advised because different laboratories use different pipelines (or different software versions). This situation means that biologists require the help of bioinformaticians even for the simplest of tasks, such as viewing their own data on a genome browser, putting these exciting techniques beyond the reach of many laboratories. Even when bioinformaticians are available, differences in priorities within collaborations can result in delays and misunderstandings that are damaging to the research effort. An optimal way to mitigate these problems is to enable biologists to perform at least basic tasks without the help of bioinformaticians by creation of user-friendly data analysis software.

Multiple standalone programs and web services are available for the analysis of NGS data. However, the majority of currently available tools have a command-line interface, perform one specific task and typically require file conversions between them. Some popular packages such as HOMER [6] or Tuxedo [7, 8] are organized into suites and include components capable of performing multiple tasks, thus solving the interoperability problem. HOMER, for example, includes tools for calling peaks, identifying motifs and performing analysis of Hi-C data. However, this excellent tool still requires the use of the command line and has limited visualization options. The commercial programs Genespring [9], Partek [10] and Golden Helix [11] can be run on regular desktop computers and allow analysis of gene expression or genetic variation. However, users have to load the data manually and store it on their desktop computers; given the sheer volume of NGS datasets, this setup makes data analysis complicated at best. Furthermore, these tools do not allow for seamless integration of multiple, published or locally produced datasets. Illumina Basespace [12] and Galaxy server [13] allow for both storage and analysis of data and have integrated viewing tools. However, they require transfer of data outside the institution (which may be prohibited by HIPAA (Health Insurance Portability and Accountability Act of 1996) regulations in some cases) and provide only limited storage space for user data. Although Galaxy provides the opportunity to run tools without using a command-line interface, users still have to manage file type conversions and select detailed parameters each time, which requires a deep understanding of each tool and file format. Absence of stable pipelines may result in inexperienced users comparing “apples to oranges”. In summary, few of the available tools provide a biologist-friendly interface, and none integrate such an interface with data storage, display and analysis.

**Fig. 1:**
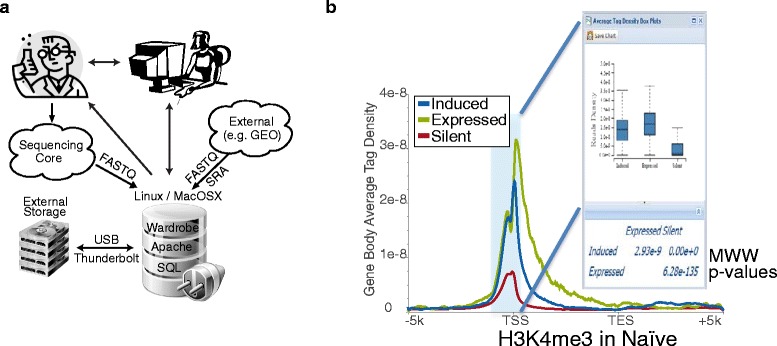
BioWardrobe overview and poising of genes in T cells. **a** The BioWardrobe server can be set up on a Linux or Mac computer attached to a storage array, typically within a local institutional network. Researchers can use BioWardrobe to upload data from a sequencing core or a public database and promptly receive quality control data, view the results in the browser, and perform some of the analysis without the assistance of bioinformaticians. Bioinformaticians can access the precomputed data in Wardrobe’s SQL database to perform further analysis. **b** The H3K4me3 average tag density profiles for the gene body in naïve T cells for genes that are expressed or silent in both naïve T helper (Thn) cells and T helper type 1 (Th1) cells and those that are induced during Thn to Th1 transition. The box plot window shows the distribution of H3K4me3 tag densities for the three gene sets within the area shaded in the plot (*Silent*, *Expressed*, *Induced*). Mann-Whitney-Wilcoxon (*MWW*) *p* values are shown below the box plot. Here and in most of the other figures the plot was produced in BioWardrobe, saved as an .svg and adjusted in Adobe Illustrator. *TES* transcription end site, *TSS* transcription start site

We therefore developed BioWardrobe, a biologist-friendly platform for integrated acquisition, storage, display and analysis of NGS data, aimed primarily at researchers in the epigenomics field. BioWardrobe features include download of raw data from core facilities or online databases (e.g., GEO), read mapping and data display on a local instance of the University of California, Santa Cruz (UCSC) genome browser [14], quality control and both basic and advanced data analysis (Fig. 1a). In basic analysis (Additional file 1: Figure S1a), automated pipelines are used to process each experiment. The pipelines are selected on the basis of biologist-friendly experimental parameters (e.g., RNA/ChIP-Seq, paired/single, genome, stranded/unstranded, antibody) and combine the tools developed by ourselves and by others (e.g., Bowtie [8], STAR [15], FASTX [16] and MACS2 [17]) with wrappers that enhance the output of original software by offering additional information (e.g., assignment of ChIP/DNase-Seq peaks to the nearest genes), provide experimentally meaningful quality controls, and display results within a web interface. The quality controls produced during basic analysis were chosen to facilitate troubleshooting of experimental procedures. Customizable advanced analysis can combine multiple experiments and includes tools for comparing gene expression (DESeq1/2 [18]) and genome occupancy (MAnorm [19]) profiles between samples or groups of samples and creating principal component analysis plots, gene lists, average tag density profiles and heatmaps using a graphical user interface (Additional file 1: Figure S1b). Incorporating additional custom scripts is facilitated by a built-in interface for the R programming language. All of the precomputed data are stored in an SQL database and can be accessed via a convenient web interface by biologists. Bioinformaticians, on the other hand, can access the data from R using a provided R library or using other programming languages with standard MySQL libraries. BioWardrobe can be run on Linux or MacOSX systems (e.g., a Mac Pro desktop). The installation package and instructions are available at [20, 21] under GNU GPL v.2. A limited-functionality demo version that contains the two datasets discussed below is available at [22].

## Results

To demonstrate the utility of the included quality controls and the ability of BioWardrobe to integrate and analyze data from various sources, we have performed re-analysis of two published datasets. The first study examined gene expression and chromatin changes during differentiation of human naïve T helper (Thn) cells into T helper type 1 (Th1) cells (SRA082670 [23]). The dataset included Helicos RNA-Seq performed in triplicates for both resting Thn cells and cells differentiated in Th1 conditions for 72 hours (Th1 cells) and H3K4me3 (histone 3 lysine 4 trimethylation) ChIP-Seq data of Th1 cells. In order to identify differentiation-related chromatin changes, we also included our own H3K4me3 ChIP-Seq data for Thn cells. After we entered sample information into the system (Additional file 1: Figure S2a), BioWardrobe downloaded the dataset and performed basic analysis (Additional file 1: Figure S3b). ChIP-Seq data demonstrated the expected percentage of reads mapped and base frequency (Additional file 1: Figure S3a–d), average tag density profiles showed high enrichment at promoters (Additional file 1: Figure S3e and f) and MACS2 identified a large number of islands (areas of enrichment), the majority of which (68–77 %) were located at promoters (Additional file 1: Figure S3g–j). However, RNA-Seq results demonstrated poor mapping to the human transcriptome, poor coverage and potential DNA and ribosomal RNA contamination (Additional file 1: Figs. S2 and S4). Keeping these problems in mind, we continued with data analysis and performed a comparison of gene expression using DESeq2. Replicates were defined, genes were grouped by common transcription start site (TSS) and differentially expressed genes were identified. These results were used to define lists of genes that were expressed or silent in both Thn and Th1 cells or induced during differentiation. Next, H3K4me3 average tag density profiles were created for these three gene lists (Fig. 1b). As demonstrated in the graphs and Mann-Whitney-Wilcoxon (MWW) statistical analysis (Fig. 1c), genes that are expressed in both Thn and Th1 cells have higher levels of H3K4me3 at their promoters than genes that are silent in both cell types. Interestingly, differentiation-induced genes had intermediate levels of this modification in naïve cells, in which they were silent, suggesting that H3K4me3 poises inducible genes for expression during differentiation.

**Fig. 2:**
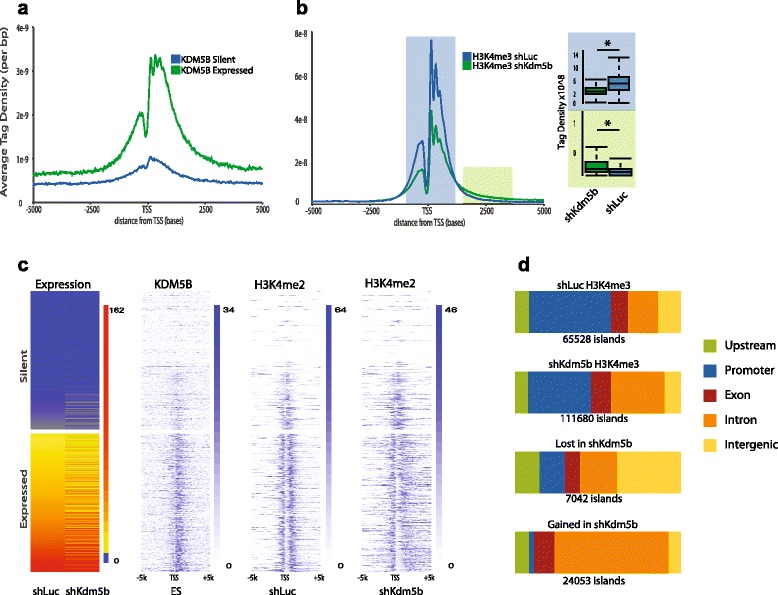
Role of KDM5B in the regulation of H3K4me3. **a** Tag density profile shows that KDM5B is recruited to promoters of expressed rather than silent genes in mouse embryonic stem cells. **b** *Kdm5b* knock-down results in increased H3K4me3 levels in the gene bodies and the corresponding loss of H3K4me3 at the TSSs of expressed genes. Box plot windows for the shaded areas are shown (*right*). * MWW p<1e-16. **c** Heatmaps show that *Kdm5b* knock-down causes spreading of H3K4me2 into the gene bodies, but not upstream, of the expressed genes. **d** Genomic distribution of H3K4me3 peaks. Top graphs show data for all peaks in control (shLuc)- and shKdm5b-expressing cells; bottom graphs show distribution for those areas where occupancy was significantly (*p* < 0.01, fold > 4) increased or decreased upon *Kdm5b* knockdown

We used a second, published dataset to examine the role of KDM5B in regulating H3K4me in mouse embryonic stem cells (GSE53093) [24]. The dataset includes RNA-Seq data for embryonic stem cells transfected with short hairpin RNA against luciferase (control, shLuc) and against *Kdm5B* (shKdm5b) RNA. ChIP-Seq was performed for KDM5B and against H3K4 dimethylation and trimethylation (H3K4me2/3) in both shLuc- and shKdm5b embryonic stem cells. Basic analysis showed good quality datasets (Additional file 1: Figure S4, right and not shown) for both ChIP-Seq and RNA-Seq. KDM5B was enriched at the promoters of expressed genes (Fig. 2a). H3K4me3 tag density profiles showed that *Kdm5b* knock-down resulted in a statistically significant redistribution of H3K4me3 tag density from promoters to the bodies of expressed genes (Fig. 2b). This was confirmed by heatmap analysis that showed spreading of both H3K4me2 (Fig. 2c) and H3K4me3 (not shown) into the gene bodies upon *Kdm5b* knockdown. Further, we compared H3K4me3 levels between individual H3K4me3 islands in shLuc- and shKDM5B-expressing cells using MAnorm. Interestingly, the majority of islands that have significantly (*p* < 0.01, fold > 4) gained H3K4me3 upon *Kdm5b* knock-down were located in gene bodies (Fig. 2d), confirming the results obtained from average tag density profiles and reported in the original publication.

In summary, we have developed a semi-automated system for storage, visualization and analysis of NGS data. BioWardrobe has been already used to analyze data in several publications [25–29]. The system can be installed on Mac or Linux computers and can provide a data analysis solution for an entire laboratory or institution.

## Materials and methods

### System overview

BioWardrobe allows users to upload, store and analyze NGS data. The workflow consists of two parts: basic and advanced analysis (Additional file 1: Figure S1). The basic analysis includes operations that do not require comparison of samples: data download, quality control, calculation of RPKMs (reads per kilobase of transcript per million reads mapped), peak identification and upload to an integrated mirror of the UCSC genome browser. Advanced analysis includes comparing gene expression or ChIP-Seq profiles between samples. BioWardrobe can work with multiple genomes (our instance currently uses human, mouse, rat, fly and frog) and additional genomes are easy to add, especially if the genome of interest is represented on the UCSC genome browser. A flexible data ownership system is implemented: though all users can see all experiments on a local mirror of the UCSC genome browser, only members of the laboratories that own the data can access and analyze datasets within the BioWardrobe web interface or download it. Laboratory-level administrators can elect to share data with other laboratories. However, trusted bioinformaticians can have access to all datasets outside of the BioWardrobe interface — e.g., via R/RStudio. We believe that this setup strikes a balance between maintaining data ownership and encouraging collaborations.

### Basic analysis

Basic analysis includes operations that are performed on a single library (Additional file 1: Figure S1a). Analysis starts by entering the experiment description into BioWardrobe. This information will be used to select the appropriate genome and analysis pipeline. Raw data can be directly downloaded by BioWardrobe via hypertext transfer protocol (http) or file transfer protocol (ftp) from core facilities or internet databases such as GEO or SRA. Compressed or uncompressed FASTQ (.fastq) or SRA (.sra) files can be used. We elected not to use prealigned BAM (.bam) files to ensure uniform alignment of samples.

For ChIP-Seq and similar experiments, reads are aligned to the genome with Bowtie [8], quality control analysis is conducted and data are summarized in a table (Additional file 1: Figure S2b). In addition to basic statistics (percentages of mapped/unmapped/non-uniquely mapped reads and average fragment length), BioWardrobe displays several other quality control measures. Base frequency plots are used to estimate adapter contamination, a frequent occurrence in low-input ChIP-Seq experiments (Additional file 1: Figure S3c). Average tag density profiles can be used to estimate ChIP enrichment for promoter proximal histone modifications (e.g., H3K4me3; Additional file 1: Figure S3e and f). The genome browser can be used to visually compare results with other experiments in the database (Additional file 1: Figure S3g and h). ChIP-Seq results are displayed on the genome browser as coverage per million reads mapped. For paired-end reads, coverage is calculated as the number of fragments covering each base pair (bp). To obtain coverage for single-read experiments, average fragment length is calculated by model-based analysis of ChIP-Seq (MACS2) [17], and individual reads are extended to this length in the 3’ direction. Islands (areas of enrichment) identified by MACS2 are displayed both on the browser (Additional file 1: Figure S3g and h) and as a table together with the nearest genes. This table can also be used to select a cutoff for significant peaks that will be used in the downstream analysis. Additionally, the fasta sequences of peaks can be obtained with the click of a button and used with third-party tools (e.g., MEME-ChIP [30]) to produce sequence logos. Use of different parameters or pipelines for different antibodies (e.g., “broad peaks” MACS2 option for H3K27me3) is possible. Additionally, users can elect to use one of the experiments in the database as an “input” control for MACS2. The distribution of the islands between genomic areas (promoters, exons, etc.) is displayed as a stacked bar graph (Additional file 1: Figure S3i and k).

For RNA-Seq analysis, reads are aligned to the genome using RNA STAR [15] provided with an appropriate annotation (e.g., RefSeq; other annotations can also be used). The quality control tab displays the number of reads aligned within and outside the transcriptome. The percentage of the reads mappable to ribosomal DNA is displayed to estimate the quality of ribosomal RNA depletion (Additional file 1: Figure S2b). Interpretation of quality control data is shown in Additional file 1: Figure S4. Data are deposited on the browser, and RPKM values are calculated for each transcript (algorithm to be described elsewhere). Depending on the application, RPKM values can be presented for each transcript or summed up for each TSS (for gene expression studies) or for each gene (for functional studies, e.g., Gene Ontology).

### Advanced analysis

If satisfied with the quality of data obtained from sequencing, a user can proceed to advanced analysis, which involves integration of information from multiple experiments. For gene expression analysis, the typical task is identifying differentially expressed genes. We elected to incorporate the DESeq1/2 algorithm [18, 31] for this purpose because it does not require recreating transcript models and does not make too many assumptions. In order to perform gene expression profiling, a user can define replicates and utilize the DESeq algorithm to calculate *p* values and fold changes for all genes. On the basis of DESeq results, lists of genes whose expression changes can be created within BioWardrobe using expression levels, fold change, or *p*/q values, as well as other parameters, and downloaded, if needed, in a table form for further analysis (e.g., gene set enrichment analysis).

The gene sets can also be used to create average tag density profiles and heatmaps within BioWardrobe (Fig. 1b). Average tag density profiles are used to compare the enrichment of histone modifications or other proteins around the TSS or the gene bodies between different gene sets. Often gene bodies are used to estimate enrichment, for instance when comparing the levels of positive marks, such as H3K4me3, between expressed and silent genes. Heatmaps provide similar information but allow comparisons of modifications between individual genes. Statistical comparison of tag densities between groups of genes using MWW test can be performed by highlighting the area of interest with a mouse (Fig. 1b, insert). All graphs can be downloaded in publication-quality scalable vector graphics (SVG) format.

For ChIP-Seq, the task is usually the identification of areas that have different levels of binding between samples. The difficulty here is that the signal-to-background ratio (enrichment) is usually slightly different between ChIP-Seq experiments; thus, several assumptions have to be made in order to compare islands of enrichment. BioWardrobe uses the MAnorm algorithm [19], which assumes that modifications do not change in the majority of areas. This allows MAnorm to adjust for differential levels of enrichment between experiments. The lists of islands, fold changes, accompanying *p* values and the nearest genes are presented in table form, and islands can be viewed in the browser with the push of a button.

### R interface

Although we sincerely believe that the set of quality control measurements and tools that we provide is the most useful, this may be a matter of personal preference. In order to allow for easy addition of custom analysis, we have incorporated R language script editing into the BioWardrobe web interface for both basic and advanced analysis steps. System administrators can add custom R scripts in the R tab, and biologists can run these scripts via the graphical web interface. In the basic analysis, customized R scripts can be run for each sample automatically or for selected samples. As an example, we have added scripts that provide the histogram of read pile-up or island length for ChIP-Seq data or gene body coverage and RPKM histogram for RNA-Seq data (Additional file 1: Figure S5). In the advanced analysis R interface, customized scripts can be provided by system administrators. Users can select records of interest via the graphical user interface and run the customized scripts as needed. As an example, we provide a principal component analysis (PCA) script that can be used for analysis of RNA-Seq data and an IDR2 script that can be used to analyze reproducibility of ChIP-Seq experiments (see [22] for output).

### Implementation

We envision BioWardrobe being installed on a dedicated server by an IT professional at a laboratory or core facility level and accessible to users via web interface using Google Chrome, Safari and Firefox browsers. The web-based interface utilizes HTML5 and JavaScript technologies. To speed up the development process, EXTJS and D3 JavaScript frameworks were used. On the server, Apache with PHP is used to process user’s requests. Linux or MacOSX native job schedulers are used to run Python pipelines. For stability, all pipelines have separate queues and process statuses. Pipeline output is stored in the SQL database with the exception of BAM files. These precomputed data are accessible by third-party software, like RStudio, that allow analysis that is not included in BioWardrobe. There are no specific hardware limitations for BioWardrobe. We have installed it on both a Linux server and Mac Pro desktop and laptop computers. An average Intel Core i7 computer with 32 gigabytes of RAM and a SATA hard disk drive (more than 100 Mb read/write speed preferred) will analyze a typical ChIP-Seq or RNA-Seq experiment within less than 2 hours.

The latest version and setup instructions are available at [20]. A limited-functionality demo version is available at [22].

## Additional file

Additional file 1:
**The following additional data are available with the online version of this paper.** Additional data file 1 contains supplementary figures S1–S5, which illustrate BioWardrobe pipelines and interface and provide examples of quality control measures implemented in BioWardrobe. (PDF 3736 kb)

## Abbreviations

bp: base pair
ChIP: Chromatin immunoprecipitation
ChIP-Seq: Chromatin immunoprecipitation sequencing
DNase-Seq: DNase I hypersensitive site sequencing
GEO: Gene Expression Omnibus
H3K4me3: Histone 3 lysine 4 trimethlation
MWW: Mann-Whitney-Wilcoxon
NGS: Next-generation sequencing
RNA-Seq: Ribonucleic acid sequencing
RPKM: Reads per kilobase of transcript per million reads mapped
sh: short hairpin
SRA: Sequence Read Archive (formerly known as Short Read Archive)
Th1: T helper type 1
Thn: naïve T helper
TSS: Transcription start site
UCSC: University of California, Santa Cruz
