# Supplementary material for: BioWardrobe: an integrated platform for analysis of epigenomics and transcriptomics data
Source: Genome Biol. 2015 Aug 7;16(1):158. doi: 10.1186/s13059-015-0720-3 (PMC4531538; doi:10.1186/s13059-015-0720-3)
Supplement: Additional file 1: — The following additional data are available with the online version of this paper. Additional data file 1 contains supplementary figures S1–S5, which illustrate BioWardrobe pipelines and interface and provide examples of quality control measures implemented in BioWardrobe. (PDF 3736 kb) [file 13059_2015_720_MOESM1_ESM.pdf]

Supplementary information

for

BioWardrobe: an integrated platform for analysis of epigenomics and transcriptomics data

Andrey V. Kartashov, M.S.[1] and Artem Barski, Ph.D. [1,2]

[1]Division of Allergy and Immunology, [2]Division of Human Genetics, Cincinnati Children's Hospital Medical Center and Department of Pediatrics, College of Medicine, University of Cincinnati, Cincinnati, OH

Correspondence: Artem.Barski@cchmc.org

Supplemetary Figure Legends

Supplementary Figure 1. BioWardrobe pipelines. (a) Basic analysis pipelines. The flow diagram shows the tools used in the basic analysis pipelines for RNA-Seq and ChIP-Seq data. **(b)** Advanced analysis allows the user to identify differentially expressed genes using DESeq, create gene lists and use these lists to generate average tag density profiles and heatmaps. Differentially bound areas can be identified with MAnorm. QC, quality control.

Supplementary Figure 2. Experiment entry form and quality control window. (a) Experiments can be entered into Wardrobe by providing basic experimental details and a link to .fastq or .sra files. BioWardrobe will download the data, select the appropriate analysis pipeline and perform quality control. **(b)** Quality control tab shows basic mapping statistics. The arrow points to the high number of reads mappable to a ribosomal DNA repeat unit, suggesting incomplete removal of ribosomal RNA from the sample. This will not affect the results, but the experiment will require more sequencing since a large number of reads will be unproductively used on ribosomal RNA. Interpretation of other quality control measures is discussed in **Supplementary Figures 3 and 4**.

Supplementary Figure 3. ChIP-Seq quality controls and interpretation. Data for H3K4me3 in Naïve (left) and Th1 cells (right) are displayed. **(a,b)** Pie charts show acceptable mapping statistics. **(c,d)** The absence of AT bias in these base frequency plots suggests enrichment of H3K4me3 in the vicinity of genic areas (also see **g**). The spiky plot in (**c**) is characteristic of adapter contamination in the library and suggests that the adapter/insert ratio during ligation needs to be decreased. This problem will not affect results, but the experiment will require more sequencing since a fraction of the reads will be unproductively used on adapter-dimers. **(e,f)** Average tag density profiles around the transcription start sites (TSSs) of all genes suggests that H3K4me3 is enriched around the TSS as expected. The experiment in (**e**) has slightly better

enrichment, whereas the experiment in (**f**) has a better resolution due to a much shorter fragment size (estimated by MACS as 287 vs. 146 for **e** and **f**, respectively). **(g,h)** Representative browser images show H3K4me3 peaks at the *PPIA* promoter. Coverage by estimated fragments (top) and islands identified by MACS are shown. **(i,j)** BioWardrobe graphs show the distribution of H3K4me3 islands between genomic areas graphically and numerically (number of islands, percentage). Note that H3K4me3 is present primarily in promoter areas.

Supplementary Figure 4. RNA-Seq quality controls and interpretation. RNA-seq data for Thn (left) and control mouse ES cells (right) are shown. **(a,b)** Pie charts show mapping statistics. In (**a**), note the poor mapping to transcriptome (high unmapped), large percentage of multi-mapped reads and reads mapping outside the annotation. These confirm insufficient ribosomal RNA removal (see also **Supplementary Figure 2b**) and suggest sample contamination with genomic DNA (or, less likely, a large amount of unannotated transcription). The latter is likely to inflate RPKM values for low expressed and non-expressed genes by a few units. **(c,d)** Base frequency plot. The general shape of the plot in (**c**) is characteristic of sequencing methods that produce reads of different lengths, such as Helicos. Also note a strong AT bias in (**c**). This bias is characteristic of genomic sequence but is not expected for transcripts, confirming the presence of DNA contamination in the sample. **(e,f)** Browser images show RNA-Seq coverage of *ACTB*/*Actb* genes. Note that the 5' end of *ACTB* in (**e**) has a higher tag density (also see (**g**)). **(g,h)** Transcript coverage plots. Note that in (**g**) coverage is biased towards the 5' end, suggesting either an inherent bias of the library construction method or RNA degradation.

Supplementary Figure 5. Example of plots produced by customizable R scripts. (a) Plots for RNA-Seq: gene body coverage and RPKM histogram. **(b)** Plots for ChIP-Seq: Rank vs. pile-up plot and Island length distribution plot.

a

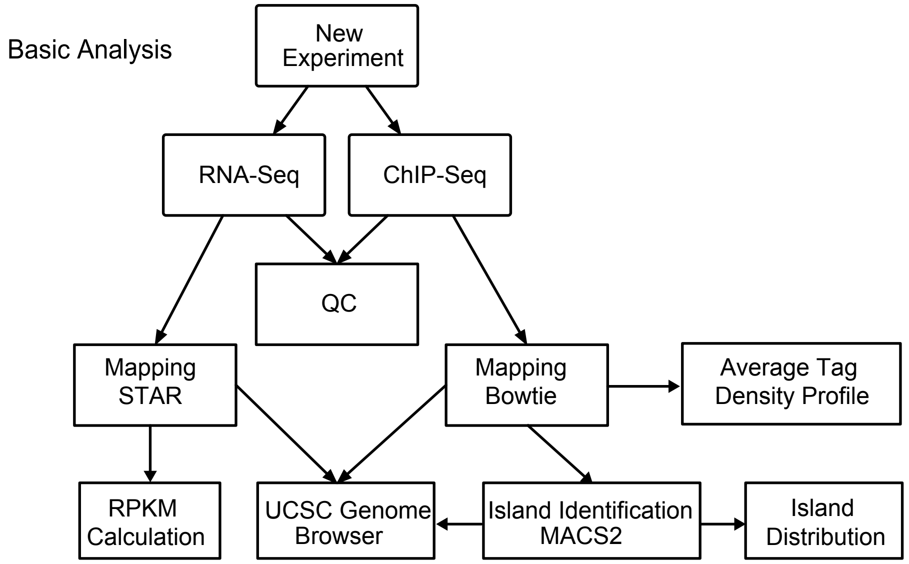

b

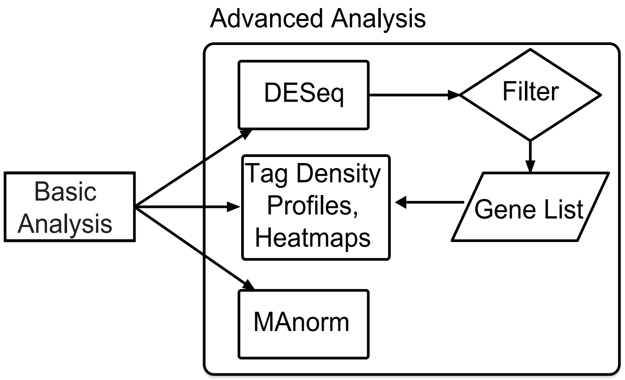

Supplementary Figure 1. BioWardrobe pipelines. (**a**) Basic analysis pipelines. The flow diagram shows the tools used in the basic analysis pipelines for RNA-Seq and ChIP-Seq data. (**b**) Advanced analysis allows the user to identify differentially expressed genes using DESeq, create gene lists and use these lists to generate average tag density profiles and heatmaps. Differentially bound areas can be identified with MAnorm. QC, quality control.

Kartashov & Barski Figure S2

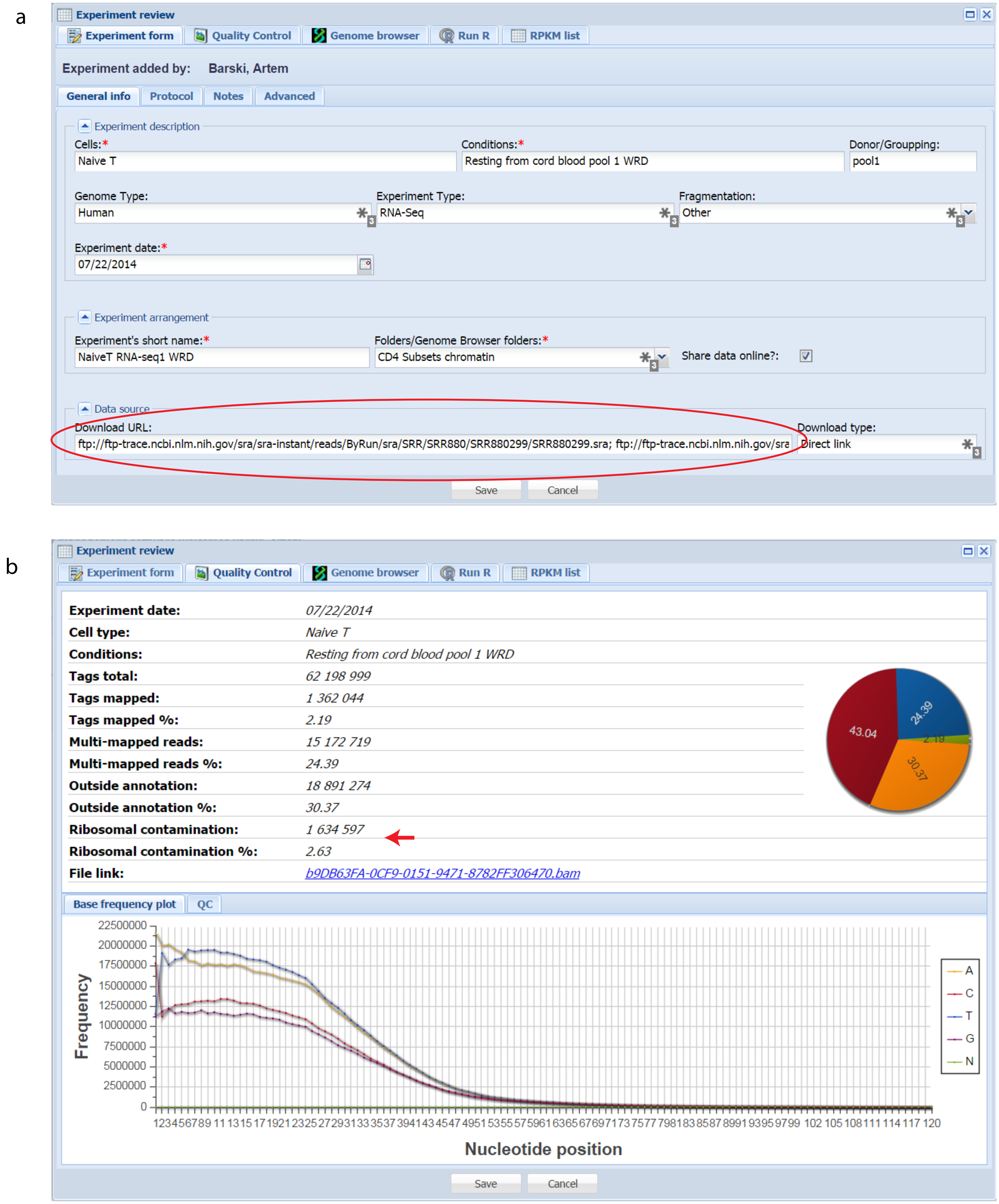

Supplementary Figure 2. Experiment entry form and quality control window. (a) Experiments can be entered into Wardrobe by providing basic experimental details and a link to .fastq or .sra files. BioWardrobe will download the data, select the appropriate analysis pipeline and perform quality control. (**b**) Quality control tab shows basic mapping statistics. The arrow points to the high number of reads mappable to a ribosomal DNA repeat unit, suggesting incomplete removal of ribosomal RNA from the sample. This will not affect the results, but the experiment will require more sequencing since a large number of reads will be unproductively used on ribosomal RNA. Interpretation of other quality control measures is discussed in Supplementary Figures 3 and 4.

Kartashov & Barski Figure S3

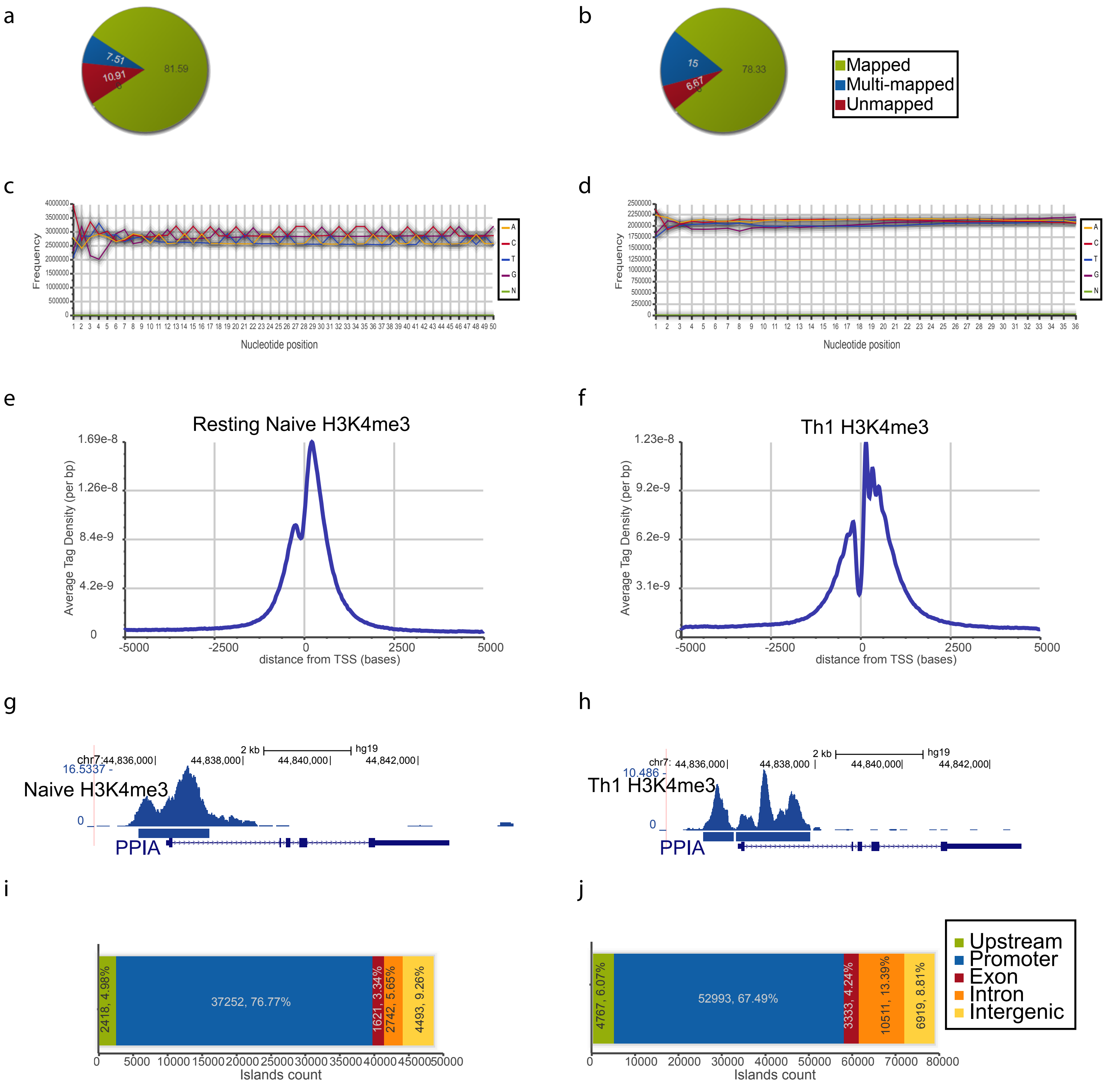

Supplementary Figure 3. ChIP-Seq quality controls and interpretation. Data for H3K4me3 in Naïve (left) and Th1 cells (right) are displayed. **(a,b)** Pie charts show acceptable mapping statistics. (**c,d)** The absence of AT bias in these base frequency plots suggests enrichment of H3K4me3 in the vicinity of genic areas (also see g). The spiky plot in (c) is characteristic of adapter contamination in the library and suggests that the adapter/insert ratio during ligation needs to be decreased. This problem will not affect results, but the experiment will require more sequencing since a fraction of the reads will be unproductively used on adapter-dimers. (**e,f**) Average tag density profiles around the transcription start sites (TSSs) of all genes suggests that H3K4me3 is enriched around the TSS as expected. The experiment in (e) has slightly better enrichment, whereas the experiment in (f) has a better resolution due to a much shorter fragment size (estimated by MACS as 287 vs. 146 for e and f, respectively). (**g,h**) Representative browser images show H3K4me3 peaks at the ACTB promoter. **(i,j)** BioWardrobe graphs show the distribution of H3K4me3 islands between genomic areas graphically and numerically (number of islands, percentage). Note that H3K4me3 is present primarily in promoter areas.

Kartashov & Barski Figure S4

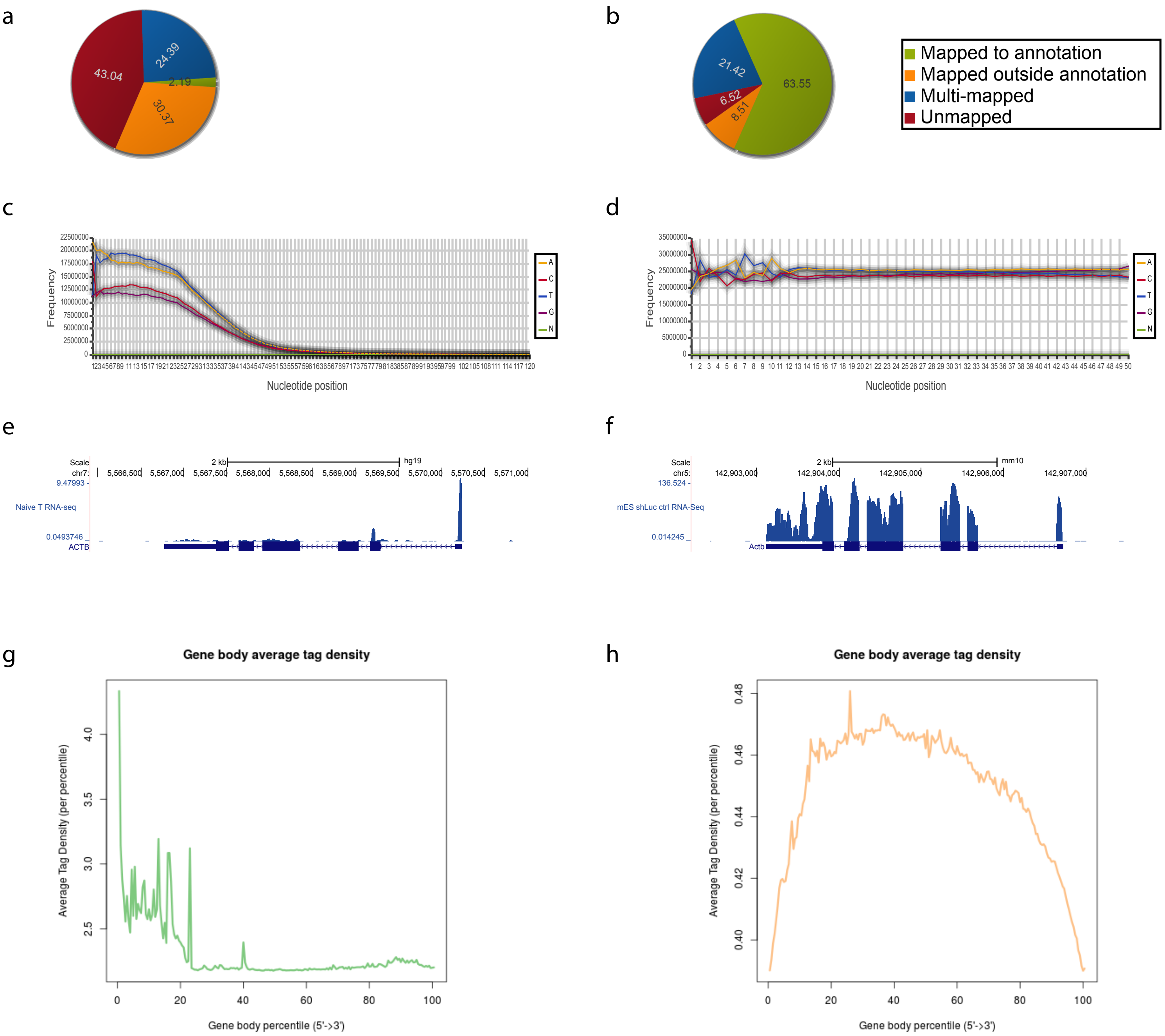

Supplementary Figure 4. RNA-Seq quality controls and interpretation. RNA-seq data for Thn (left) and control mouse ES cells (right) are shown. (a,b) Pie charts show mapping statistics. In (a), note the poor mapping to transcriptome (high unmapped), large percentage of multi-mapped reads and reads mapping outside the annotation. These confirm insufficient ribosomal RNA removal (see also Supplementary Figure 2b) and suggest sample contamination with genomic DNA (or, less likely, a large amount of unannotated transcription). The latter is likely to inflate RPKM values for low expressed and non-expressed genes by a few units. (c,d) Base frequency plot. The general shape of the plot in (c) is characteristic of sequencing methods that produce reads of different lengths, such as Helicos. Also note a strong AT bias in (c). This bias is characteristic of genomic sequence but is not expected for transcripts, confirming the presence of DNA contamination in the sample. (e,f) Browser images show RNA-Seq coverage of ACTB/Actb genes. Note that the 5′ end of ACTB in (e) has a higher tag density (also see (g)). (g,h) Transcript coverage plots. Note that in (g) coverage is biased towards the 5′ end, suggesting either an inherent bias of the library construction method or RNA degradation.

Kartashov & Barski Figure S5

a

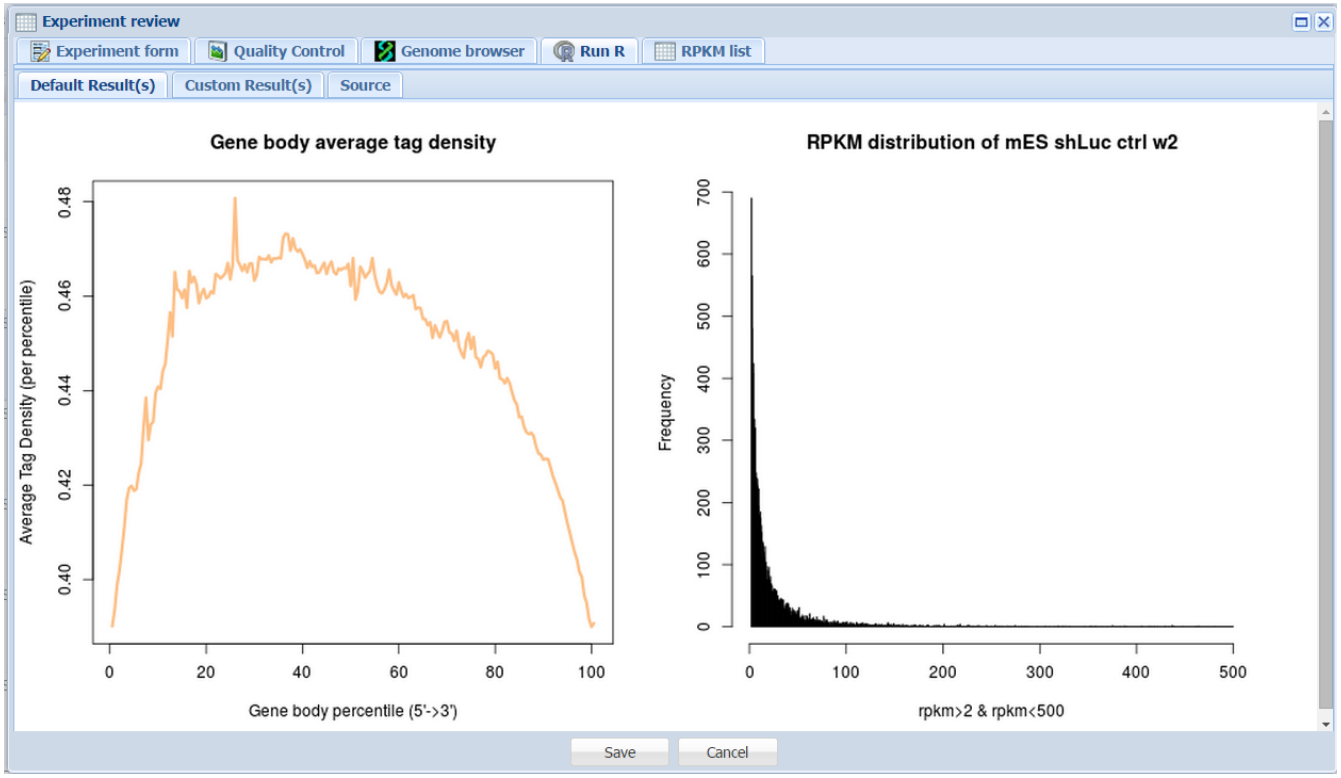

b

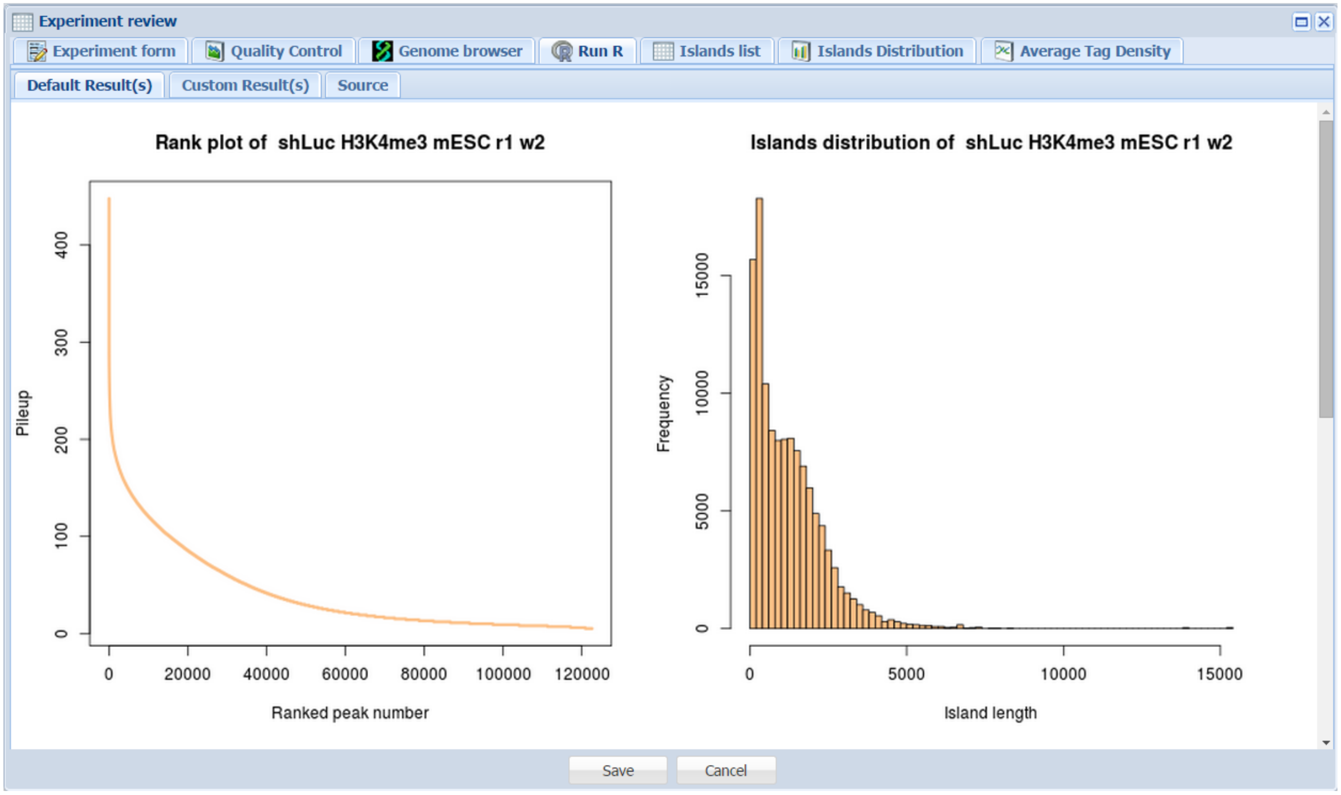

Supplementary Figure 5. Example of plots produced by customizable R scripts. (a) Plots for RNA-Seq: gene body coverage and RPKM histogram. **(b)** Plots for ChIP-Seq: Rank vs. pile-up plot and Island length distribution plot.
